# Alisol A Suppresses Proliferation, Migration, and Invasion in Human Breast Cancer MDA-MB-231 Cells

**DOI:** 10.3390/molecules24203651

**Published:** 2019-10-10

**Authors:** Chenghua Lou, Xintong Xu, Yan Chen, Huajun Zhao

**Affiliations:** School of Pharmaceutical Sciences, Zhejiang Chinese Medical University, Hangzhou 310053, China; inclovers@163.com (X.X.); 13735558952@163.com (Y.C.)

**Keywords:** alisol A, breast cancer, proliferation, autophagy, metastasis

## Abstract

Natural products are a precious source of promising leads for the development of novel cancer therapeutics. Recently, triterpenoids in Alismatis rhizoma has been widely demonstrated for their anti-cancer activities in cancer cells. In this study, we examined the inhibitory effects of alisol A in human breast cancer cells. We demonstrated that alisol A exhibited significant anti-proliferative effects in MDA-MB-231 cells and this response was related to autophagy induction. Alisol A-induced autophagy was supported by the triggered autophagosome formation and increased LC3-II levels. Interestingly, autophagy inhibitor 3-MA significantly reversed the cytotoxic effects induced by alisol A. Meanwhile, alisol A-induced autophagy was significantly inhibited by 3-MA in MDA-MB-231 cells. Cell cycle analysis revealed that alisol A arrested the cell cycle at G0/G1 phase. The expression level of cell cycle regulatory proteins cyclin D1 was significantly down regulated. In addition, the suppression of NF-κB and PI3K/Akt/mTOR pathways in MDA-MB-231 cells was observed. Furthermore, alisol A significantly suppressed the migration and invasion of MDA-MB-231 cells by inhibiting the expression levels of MMP-2 and MMP-9. Taken together, our results demonstrated that alisol A could inhibit the proliferation and metastasis of MDA-MB-231 cells. It could be a promising agent for breast cancer therapy.

## 1. Introduction

Breast cancer is the most frequently diagnosed cancer and the leading cause of cancer death in women’s worldwide [1]. Every year, almost 1.7 million women are diagnosed with breast cancer [2]. Treatment options to breast cancer include surgery, radiation therapy, chemotherapy, and targeted therapies under clinical trials [3]. Although tremendous progresses have been seen in the treatment of breast cancers, controversies remain. Every year, more than 522,000 patients worldwide die from this disease [2]. Therefore, the development of new anti-cancer agents for breast cancer is important to reduce the mortality caused by this disease.

Natural products are a precious source of promising leads for the development of novel cancer therapeutics, due to their potential effectiveness and low toxicity profiles [4,5,6]. Alismatis rhizoma is the rhizome of *Alisma orientale* (Sam.) Juzep, an aquatic plant, belonging to the Alismataceae family, which is widely distributed in China, Korea, and Japan [7]. In China, it has been widely used as a folk diuretic and hypolipidemic agents for more than a thousand years, and has been used for the treatment of dysuria, hypertension, edema, and urinary tract infections [7,8,9]. Modern pharmacological investigations have demonstrated the diuretic, anti-hypertensive, anti-cancer, hypoglycemic, and anti-atherosclerotic activities of Alismatis Rhizoma [7,10,11,12,13,14,15]. 

**Figure 1:**
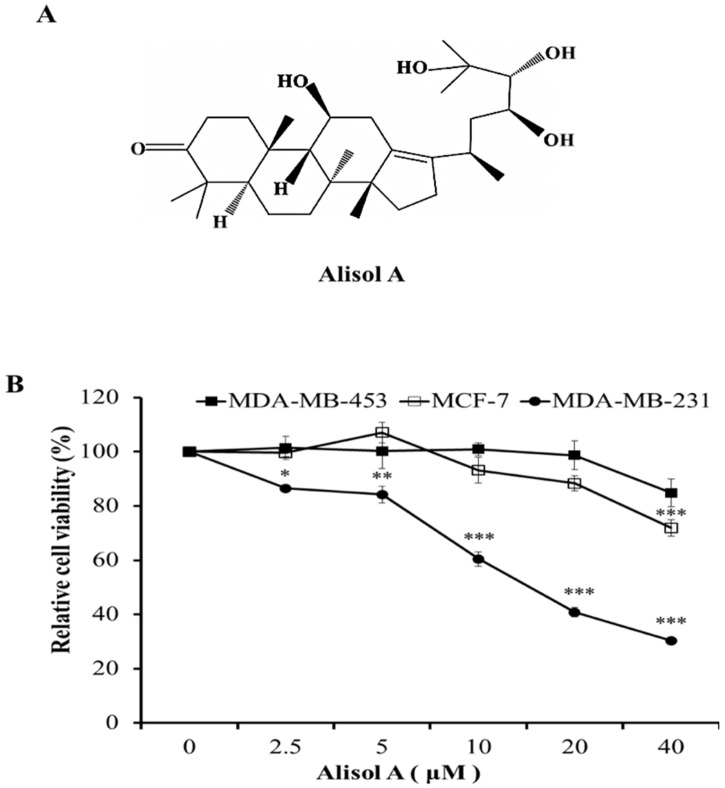
Effects of alisol A on cell viability in human breast cancer cells. (**A**) The chemical structure of alisol A. (**B**) Effects of alisol A on cell viability in MDA-MB-231, MDA-MB-453, and MCF-7 cells. Cells were treated with different concentrations of alisol A for 24 h. Then, cell viability was quantified by the MTT assay. Data represent the mean ± S.D of at least three independent experiments. * *p* < 0.05, ** *p* < 0.01, *** *p* < 0.001.

The chemical constituents of Alismatis rhizome mainly consist of triterpenoids, polysaccharides, sesquiterpenes, diterpenes, and essential oil [16]. Alisol A (Figure 1A), belonging to protostane-type tetracyclic triterpenoid, serves as one of the main components in Alismatis Rhizoma. However, there is little information concerning its anti-cancer activity. In this study, we investigated the anti-cancer activity of alisol A in human breast cancer cells and attempted to elucidate its possible molecular mechanism. 

## 2. Material and Methods

### 2.1. Cell Culture and Reagents

MDA-MB-231, MCF-7, and MDA-MB-453 cell lines were purchased from the Cell Bank of the Institute of Biochemistry and Cell Biology, China Academy of Sciences (Shanghai, China) and stored in liquid nitrogen. Cells were cultured in DMEM culture medium (Gibco, Grand Island, NY, USA) containing 10% fetal bovine serum (FBS, Gibco, USA), 100 U/mL penicillin G, 2.5 μg/mL amphotericin B, and 100 μg/mL streptomycin (complete medium) at 37 °C with 5% CO_2_ in a humidified atmosphere. 

Alisol A was purchased from MedChemExpress (Monmouth Junction, NJ, USA) (The chemical structure is shown in Figure 1A). 3-(4,5-dimethyl-2-thiazolyl)-2,5-diphenyl-2*H*-tetrazolium bromide (MTT), acridine orange (AO), 3-MA, and dimethyl sulfoxide were purchased from Sigma-Aldrich (St. Louis, MO, USA). Fetal bovine serum, Propidium iodide (PI)/RNase staining kit, and Annexin V-FITC/7AAD kit were purchased from Becton Dickinson (San Diego, CA, USA). Antibodies against caspase-3 (#9662), cleaved caspase-3 (Asp175, #9664), cyclin D1 (#2978), caspase-9 (#9508), cleaved caspase-9 (Asp330, #9501), STAT3 (#30835), NF-κB (#8242), p-NF-κB (#8242), caspase-8 (#4790), LC3 (#3868), MMP-2 (#4022), MMP-9 (#13667), mTOR (#2983), p-mTOR (#5536), p70S6K (#9202), Akt (#4685), p-Akt (#4060), Erk (#4695), p-Erk (#9101), β-tubulin (#2128), and horseradish peroxidase-conjugated secondary antibodies were purchased from Cell Signaling Technologies (Danvers, MA, USA). p21 (ab18209) were purchased from Abcam (Cambridge, UK).

### 2.2. Cell Viability Assay

The efficiency of alisol A on the proliferation of MDA-MB-231, MDA-MB-453, and MCF-7 cells was evaluated by the MTT assay, and the viability of cell numbers was correlated with the production of formazan. Cells were seeded in 96-well culture plates (5 × 10^3^ cells/well), respectively. After treatment with different concentrations of alisol A for 24 h, 20 μL MTT solution (5 mg/mL) was added. Cells were incubated at 37 °C in a 5% CO_2_ humidifier incubator for another 4 h. Formed formazan crystals were dissolved in 100 μL DMSO and the absorbance was measured at 570 nm on a microplate reader (BIO-RAD, Hercules, CA, USA). 

### 2.3. Annexin V-FITC/PI Double Staining Assay

Quantification of apoptotic cells was performed using an Annexin V-FITC Apoptosis Detection Kit according to manufacturer’s instructions. Briefly, MDA-MB-231 cells were plated in 6-well plates and then treated with different concentrations (0, 10, 20, and 40 μM) of alisol A for 24 h. Cells were harvested and washed twice with ice-cold PBS. The collected cells were then re-suspended in 500 μL of 1× binding buffer, 5 μL Annexin V-FITC, and 5 μL of propidium iodide were added and incubated for 15 min at room temperature in the dark. The number of apoptotic cells were quantified by flow cytometer and data analyzed by CellQuest software (version 5.1, BD Biosciences, Franklin Lakes, NJ, USA).

### 2.4. Acridine Orange Staining 

Acridine orange (AO) (Sigma-Aldrich Co.) was used to evaluate and quantify the formation of acid vesicular organelles (AVOs) by fluorescence microscopy. AO is an acidotropic fluorescent dye that stain DNA and cytoplasm bright green and when protonated in the presence of acid compartments it fluorescences bright red. Cells were plated in 6-well plates (3 × 10^5^ cells/well) and treated with different concentrations of alisol A for 24 h, the cells were washed twice with ice-cold PBS, and incubated with AO, which was added at a final concentration of 1 μg/mL for 15 min at 37 °C. Subsequently, cells were washed three times with ice-cold PBS and then observed under a fluorescence microscope (Nikon, Tokyo, Japan). Then, the fluorescence intensity was quantitatively analyzed using Image-Pro Plus 6.0 (Media Cybernetics, Inc., Rockville, MD, USA).

### 2.5. Cell Cycle Analysis

Cells (3 × 10^5^ cells/well) were grown in 6-well plates and treated with alisol A (0, 10, 20, and 40 μM) for 24 h. Cells were washed and collected after trypsinisation. Then, cells were fixed with 70% alcohol and allowed to cool for overnight at −20 °C. After fixation, cells were harvested and stained with PI/RNase (0.5 mL/test, 1 × 10^6^ cells) for 15 min at room temperature before analysis. Samples were subsequently analyzed by flow cytometer (Guava Technologies, Merck Drugs & Biotechnology, Darmstadt, Germany) and DNA content was quantified using ModFit LT 5.0 software (Verity Software House, Topsham, ME, USA). For flow cytometric analysis, at least 10,000 events per sample were recorded.

### 2.6. Wound Healing Assay

Wound healing assay was used to assess the effects of alisol A on cell migration. MDA-MB-231 cells were seeded into 24-well plate (2 × 10^5^ cells/well) and cultured until confluent. The monolayer cells were scratched with a sterile 1000 μL pipette tip followed by washing with PBS to remove the floating cells. Cells were treated with alisol A for 24 h. Scratched areas were photographed (magnification 40×) at zero hour and then subsequently again 24 h later to assess the degree of wound healing. The migration rate was calculated according to the following equation: Wound closure % = 1 − (wound area at t_24_/wound area at t_0_) × 100%, where t_24_ is the time after wounding and t_0_ is the time immediately after wounding.

### 2.7. Transwell Migration and Invasion Assays

Transwell migration assay was used to evaluate the in vitro anti-migratory effects of alisol A. Briefly, MDA-MB-231 cells were incubated in the presence or absence of alisol A for 24 h. After trypsinization, 1 × 10^5^ cells suspended in 0.1% (*v*/*v*) BSA medium were placed in the upper chamber of 8 μm pore size Transwells (24-well, Millipore) and incubated for 18 h at 37 °C under 5% CO_2_. For the invasion assay, the upper surface of the Transwell membrane was coated with 1 μg matrigel. Cells (2 × 10^5^) (incubated in the presence or absence of alisol A for 24 h) in 0.1% (*v*/*v*) BSA medium were placed in the upper part of the Transwell membrane and allowed to migrate for another 24 h. For both the migration and invasion assay, the unmigrated cells were removed from the upper surface of the membrane and the migrated cells on the lower surface of the membrane were fixed in 100% methanol and stained with hematoxylin and eosin. Migration was determined by counting the cell number with a microscope at ×100 magnification. Five visual fields were chosen randomly and the average number of migrating cells in the five fields was taken for each group.

### 2.8. Gelatin Zymography

The enzymatic activities of MMP-2 and MMP-9 were assayed by gelatin zymography in the absence of serum. The culture supernatants from alisol A-treated cultures were collected and centrifuged to remove debris. After that, the media was concentrated by centrifugal filters (Amicon^®^ Ultra, Millipore, Cambridge, MA, USA). The samples thus prepared were electrophoresed on 7.5% polyacrylamide gel containing 0.1% SDS and 0.1% gelatin at 4 °C. After electrophoresis, gels were washed twice with a rinsing buffer at room temperature for 1 h to remove SDS, then incubated with the incubation buffer for 42 h at 37 °C and stained with a staining solution. The locations of gelatinolytic enzymes were visualized as clear bands on the blue background. The bands were scanned by an image scanner and quantified by Image J software (Version 1.8, Softonic, Barcelona, Spain).

### 2.9. Western Blotting

MDA-MB-231 cells were pretreated with varying concentrations of alisol A. After 24 h, cells were washed immediately with pre-cold PBS twice on ice and lysed in RIPA buffer containing phosphatase inhibitors and protease inhibitors. The cell lysates containing equal amounts of total proteins were separated by SDS-PAGE and transferred to PVDF membrane, blocked with 5% nonfat milk at room temperature for 1 h, and incubated with the respective specific primary antibodies overnight at 4 °C. The membranes were washed three times with Tris-buffered saline-5% Tween 20 (TBST) solution and incubated with a horseradish peroxidase-conjugated secondary antibody at room temperature for 2 h. Chemiluminescent detection was performed by ECL (BIO-RAD, USA). 

### 2.10. Statistical Analysis

All data are expressed as mean ± S.D of three independent experiments. Statistical significance was analyzed using Student’s t-test. The criterion of statistical significance was * *p* < 0.05; ** *p* < 0.01; *** *p* < 0.001.

## 3. Results

### 3.1. Effects of Alisol A on Cell Viability in Human Breast Cancer Cells

To determine the effects of alisol A on the growth of human breast cancer cells, the cytotoxic effects were measured by MTT assay. Breast cancer is a heterogeneous disease with high degree of diversity based on histology, cellular origin, metastatic potential, therapeutic response, and clinical outcome [17]. Generally, there are three identified types: HER2 (+), ER/PR (+), and TNBC (defined by the lack of ER, PR, and HER2 in breast cancer cells) breast cancer cells [18]. In the present study, MDA-MB-231 (TNBC), MCF-7 (ER/PR (+)) and MDA-MB-453 (HER2 (+)) cell lines were used. Cells were treated with different concentrations of alisol A for 24 h. As shown in Figure 1B, alisol A significantly inhibited the growth of MDA-MB-231 cells in a concentration-dependent manner. However, alisol A did not show obvious cytotoxic effects on MCF-7 and MDA-MB-453 cells. Therefore, MDA-MB-231 cells were considered as an in vitro model for further study. 

### 3.2. Effects of Alisol A on Induction of Cell Apoptosis

**Figure 2:**
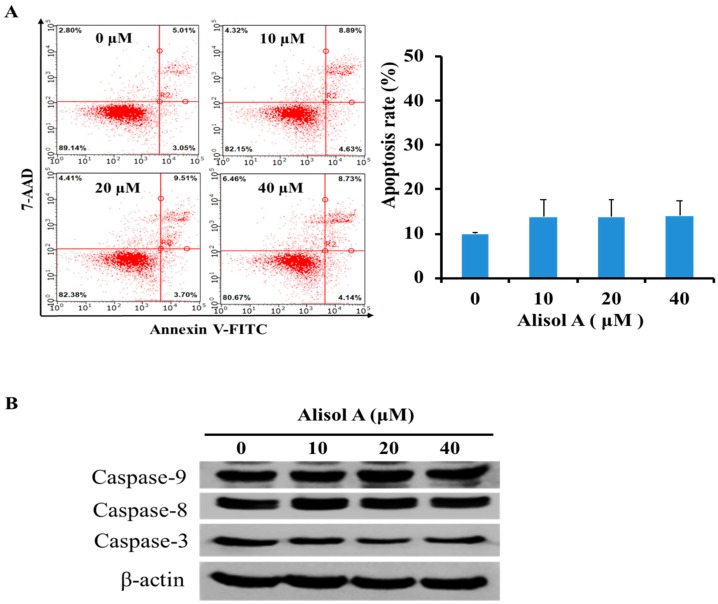
Effects of alisol A on induction of cell apoptosis. (**A**) Quantification of apoptotic cells was performed by flow cytometer. MDA-MB-231 cells were treated with different concentrations of alisol A for 24 h. Cells were stained with Annexin-V-FITC/7AAD according to the manufacturer’s instructions. (**B**) Effects of alisol A on the expression of caspases in the MDA-MB-231 cells. The cells were treated with alisol A for 24 h. The cell lysates were collected and subjected to Western blotting analysis. Results are representative of at least three independent experiments showing similar results.

To determine whether the growth inhibitory effects of alisol A were associated with the induction of apoptosis, Annexin V-FITC/PI double staining was used as a criterion to distinguish apoptotic cells by flow cytometry analysis. As shown in Figure 2A, alisol A treatment for 24 h did not significantly increase the number of apoptotic cells in MDA-MB-231 cells. The percentage of apoptotic cells was increased from 9.90 ± 0.34% (0 µM) to 14.03 ± 3.36% (40 µM). Meanwhile, we did not observe significant activation of cleaved-caspases (caspase-3, caspase-8, and caspase-9) in MDA-MB-231 cells by Western blotting analysis with alisol A treatment (Figure 2B). These results indicated that the induction of apoptotic cell death was not the potential mechanism of alisol A against MDA-MB-231 cancer cells.

### 3.3. Effects of Alisol A on Induction of Autophagy

**Figure 3:**
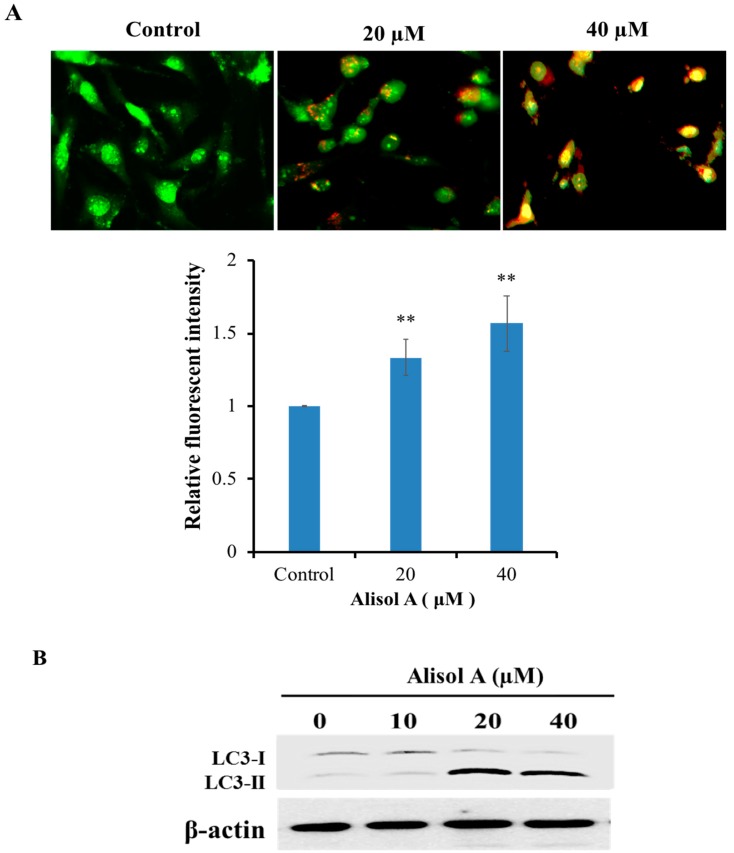
Effects of alisol A on induction of autophagy. (**A**) Autophagy in alisol A-treated MDA-MB-231 cells were stained with AO and examined under a fluorescence microscope. Quantitation of formation of autophagolysosomes in alisol A-treated cancer cells was performed. (**B**) Western blotting analysis of the protein expression of LC3 in MDA-MB-231 cells treated with alisol A for 12 h. Data are expressed as mean ± S.D of at least three independent experiments. ** *p* < 0.01.

**Figure 4:**
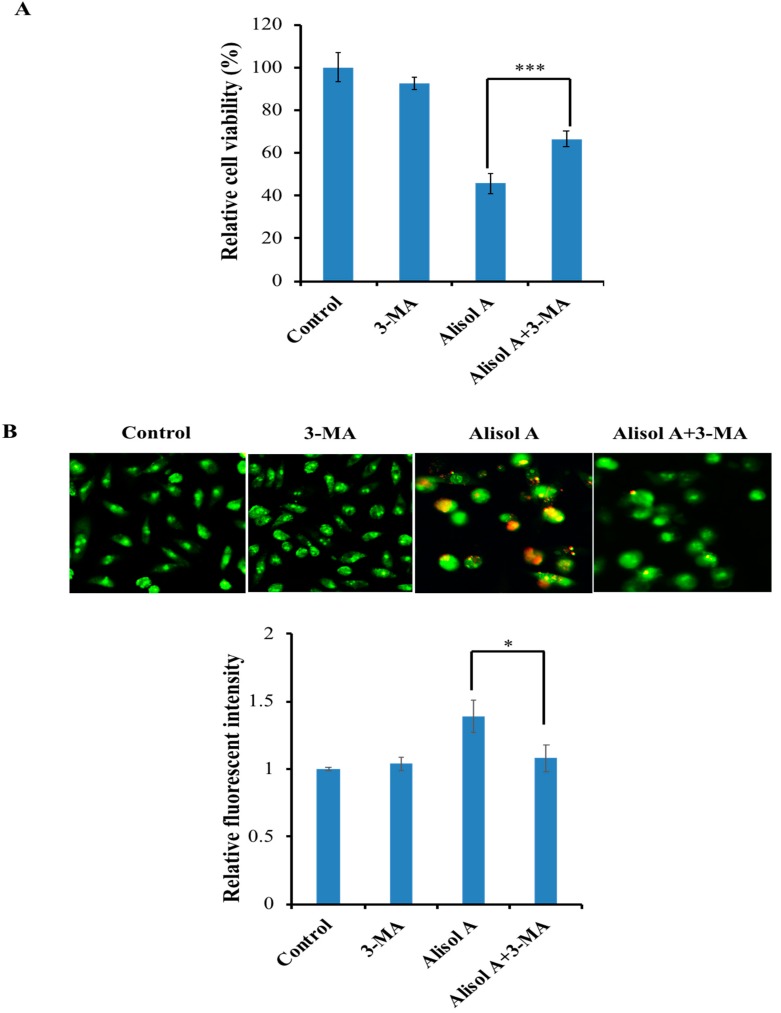
Effects of 3-MA on alisol A-induced autophagy. (**A**) Autophagy inhibitor 3-MA reversed the cytotoxic effects induced by alisol A. MDA-MB-231 cells were pre-incubated with or without 3-MA (5 mM) for 2 h and then incubated with alisol A (20 µM) for another 24 h, after which the cells were subjected to MTT assay. (**B**) Autophagy in alisol A-treated MDA-MB-231 cells were inhibited by 3-MA. Quantitation of formation of autophagolysosomes in treated cancer cells was performed. Data are expressed as mean ± S.D of at least three independent experiments. * *p* < 0.05, *** *p* < 0.001.

Numerous studies have shown that autophagy functions as a tumor suppressor through removing aberrant proteins and organelles in tumorigenesis. To understand whether autophagy played an important role in alisol A induced cell death, we examined the formation of autophagic vacuoles with AO staining. AO, an indicator of autophagy, is widely used to evaluate and quantify the formation of acid vesicular organelles (AVOs). In acid compartments, such as lysosomes and autolysosomes, the fluorescence of AO switches from green to red color. As shown in Figure 3A, we observed an increasing red fluorescence with the increasing concentration of alisol A, while the control cells primarily exhibited green fluorescence. Meanwhile, we found that alisol A also induced the accumulation of LC3-II (Figure 3B), an autophagy-related, ubiquitin-like modifier, regarded as an autophagosomal marker in mammals cells. Interestingly, the autophagy inhibitor 3-MA could significantly reverse the cytotoxic effects induced by alisol A (Figure 4A). Meanwhile, 3-MA significantly inhibited alisol A-induced autophagy in MDA-MB-231 cells (Figure 4B). These results indicated that the induction of autophagy could be a potential mechanism of alisol A against MDA-MB-231 cells.

### 3.4. Effects of Alisol A on Cell Cycle Arrest

**Figure 5:**
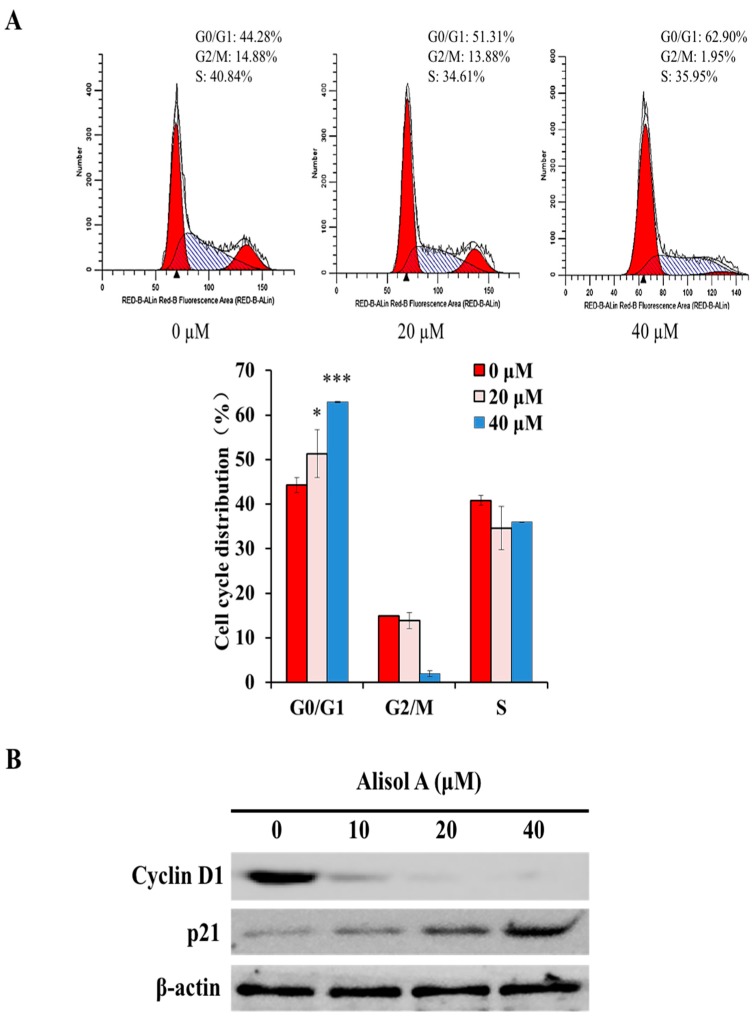
Effects of alisol A on cell cycle in MDA-MB-231 cells. (**A**) MDA-MB-231 cells were treated with alisol A for 24 h, fixed in 70% ethanol at 4 °C overnight, and stained with propidium iodide (PI). Cell cycle distribution was assessed by flow cytometry. Quantified histograms display the effects of alisol A on cell cycle distribution. (**B**) The expression of cell cycle-related proteins, Cyclin D1 and p21 in MDA-MB-231 cells were analyzed by Western blotting. Data are presented as the mean ± S.D of three independent experiments. * *p* < 0.05, *** *p* < 0.001.

To investigate whether alisol A modulated the cell cycle in MDA-MB-231 cells, cells were treated with different concentrations of alisol A for 24 h. As shown in Figure 5A, the percentage of cells in the G0/G1 phase was significantly increased. Consistent with the above results, Western blotting results showed that the expression of cell cycle regulatory protein Cyclin D1 was significantly down-regulated, while the expression of p21 was up-regulated (Figure 5B). These results indicated that the cell cycle arrest in G0/G1 phase might be one of the mechanisms of alisol A against breast cancer cells.

### 3.5. Effects of Alisol A on Autophagy Related Signaling Pathways

**Figure 6:**
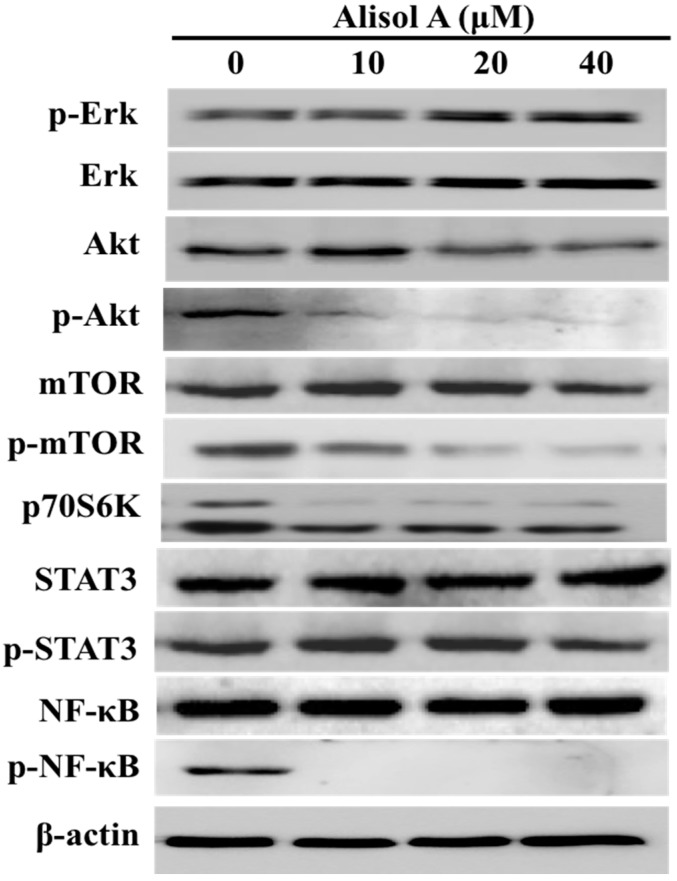
Effects of alisol A on autophagy related signaling pathways. The cells were treated with alisol A for 24 h. The cell lysates were collected and subjected to Western blotting analysis to detect the expression of proteins in cancer cells. Results are representative of at least three independent experiments showing similar results.

To further explore the molecular mechanism of alisol A on induction of autophagy in MDA-MB-231 cells, we assessed its effects on the autophagy related signaling pathways. As shown in Figure 6, our results demonstrated that the expression of p-Akt, p-mTOR, p70S6K, and p-NF-κB was significantly down-regulated with the treatment of alisol A. However, alisol A-treatment did not show obvious effects on Erk and STAT3 signaling pathways (Figure 6). Those results indicated that the suppression of NF-κB and PI3K/AKT/mTOR pathways in MDA-MB-231 cells might contribute to alisol A-induced cell death.

### 3.6. Effects of Alisol A on Cell Migration and Invasion

**Figure 7:**
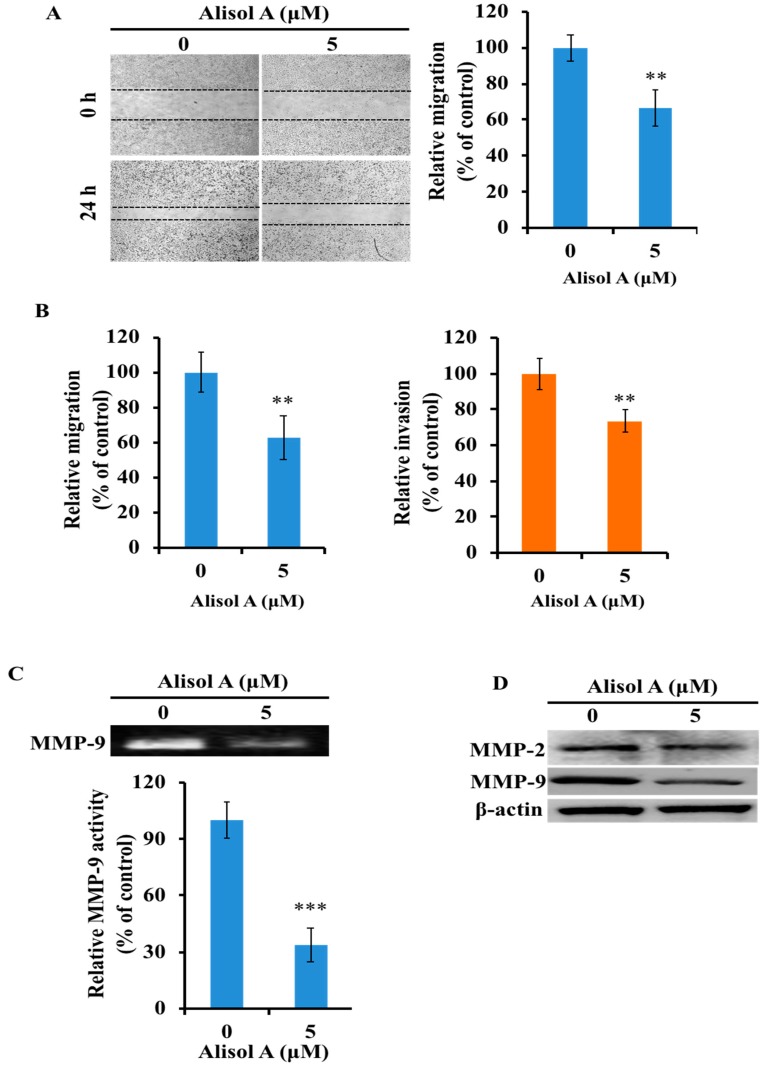
Effects of alisol A on cell migration and invasion. (**A**) The effects of alisol A on cell migration was measured by wound healing assay. (**B**) Transwell migration (left) and invasion (right) assay was used to evaluate the in vitro anti-metastatic effects of alisol A. (**C**) Effects of alisol A on MMP-9 activity by gelatin zymography assay. (**D**) Effects of alisol A on MMP-2 and MMP-9 protein expression in MDA-MB-231 cells. Data are presented as the mean ± S.D of three independent experiments. ** *p* < 0.01, *** *p* < 0.001.

To further determine whether alisol A could suppress the migration of MDA-MB-231 cells, mechanical wounds were introduced into confluent monolayers, and wound closure was measured by microscopy. As previously demonstrated, alisol A at a concentration of 5 μM did not show significant inhibitory effects on the viability of the MDA-MB-231 cells. Therefore, this concentration was selected for further evaluation of the anti-invasion and anti-migration effects of alisol A. As shown in Figure 7A, alisol A significantly inhibited the wound closure at a dosage of 5 µM (*p* < 0.01). Compared to the control groups, the wound closure rate was reduced to 66.67 ± 9.89%. Moreover, Transwell migration assay also demonstrated the anti-migration effects of alisol A (Figure 7B left). With the treatment of alisol A (5 µM), the cell migration rate was reduced to 62.77 ± 12.33%. To further confirm the activity of alisol A on cell invasion, Transwell invasion assay was performed to evaluate the anti-invasion effects of alisol A on MDA-MB-231 cells. As shown in Figure 7B (right), the results showed that alisol A significantly inhibited cell invasion. These results suggested that alisol A was effective in reducing the migration and invasion of MDA-MB-231 cells.

### 3.7. Effects of Alisol A on MMP-2 and MMP-9 in MDA-MB-231 Cells

To determine whether the inhibitory effects of alisol A on the invasion of MDA-MB-231 cells were related to the MMPs activity, gelatin zymography assay was performed to examine the activity of MMPs. As shown in Figure 7C, alisol A-treated MDA-MB-231 cells showed a significant reduction in the activity of MMP-9 (*p* < 0.001). Furthermore, Western blotting assay was performed to examine the expression level of MMP-2 and MMP-9 in cancer cells after treatment with alisol A. As shown in Figure 7D, the expression level of MMP-2 and MMP-9 was significantly down-regulated. These results demonstrated that the down regulation of MMP-2 and MMP-9 in MDA-MB-231 cells was related to the anti-metastatic effects of alisol A.

## 4. Discussion

Alismatis rhizoma is a common traditional herbal medicine in China. Recently, chemical constituents in Alismatis rhizoma have been widely demonstrated for their anti-cancer activities. According to the reports, alisol B and alisol B 23-acetate were demonstrated to suppress cancer cells by cell cycle arrest, apoptosis induction, and metastasis inhibition [11,19,20]; the extract and compounds from Rhizoma Alismatis could reverse the multidrug resistance in cancer cells [21,22]. In the present study, we evaluated the anti-cancer activity of alisol A in human breast cancer cells. The experimental results demonstrated that alisol A inhibited the proliferation of MDA-MB-231 cells through autophagy induction (Figure 3 and Figure 4) and cell cycle arrest at G0/G1 phase (Figure 5). Moreover, alisol A also exhibited significant anti-metastatic activities in vitro (Figure 7).

Apoptosis in cancer cells is a promising treatment method in cancer therapy. In general, drug-induced apoptosis is one major mechanism of action for the treatment of cancer, and various signaling pathways are involved in the process [23,24]. Interestingly, in our study, alisol A-treatment did not significantly increase the number of apoptotic cell death in MDA-MB-231 cells (Figure 2A), and the activation of cleaved-caspases (caspase-3, caspase-8, and caspase-9) was also not observed (Figure 2B), which indicated that the induction of apoptotic cell death is not the main mechanism of alisol A against MDA-MB-231 cancer cells.

Autophagy, which plays a critical role in the control of cell proliferation, differentiation, and cell death, has been extensively characterized in the past decades [25,26]. Recent studies have demonstrated that autophagic activity is elevated in different types of cancers and is considered as a therapeutic target in several clinical trials [27,28]. The role of autophagy in cancer is controversial. It is known as a double-edged sword for cancer. On the one hand, autophagy can protect cancer cells from apoptosis and promotes metastasis [29,30]. On the other hand, it can induce autophagic cell death, impede metastasis, inhibit cell proliferation, and even enhance chemosensitivity [31,32,33]. Interestingly, we demonstrated the autophagy induction in alisol A-treated MDA-MB-231 cells by AO staining (Figure 3A). In autophagosome formation, LC3 is considered as a specific marker of autophagy. It is cleaved to form LC3-I when autophagy is induced, then conjugated and further processed to form LC3-II [34,35], which is involved in the final membrane fusion steps as well as in the localization of degradation targets to the autophagosome [36,37]. In the present study, the expression level of LC3-II was significantly up-regulated (Figure 3B). Notably, autophagy inhibitor 3-MA significantly reversed the cytotoxic effects induced by alisol A (Figure 4), which indicated that autophagy induction could be a potential mechanism of alisol A against MDA-MB-231 cells.

The PI3K/Akt/mTOR signaling pathway is an important intracellular mediator, which is considered as a classic negative regulator of autophagy [38,39]. Studies have demonstrated disorders of the PI3K/Akt/mTOR signaling pathway in many tumors. In cancer cells, PI3K/Akt activity is increased, which activates mTOR complex via phosphorylation and decreases the feedback activation of p70S6k1/mTOR complex [40]. mTOR, a critical regulator of autophagy induction, functions by inhibiting the downstream molecular complex ULK1 to negatively regulate autophagy levels [38]. In this study, the expression levels of p-Akt, p-mTOR, and p70S6K were significantly down-regulated after alisol A treatment (Figure 6), suggesting that alisol A could suppress the PI3K/Akt/mTOR signaling pathway in MDA-MB-231 cells. On the other hand, nuclear factor-κB (NF-κB) is well-established to play a critical role in the control of cell proliferation and oncogenesis [41,42]. Recent studies have demonstrated that some anti-cancer agents show anti-cancer effects by induction of autophagy through suppressing NF-κB activation [43]. Interestingly, the expression of p-NF-κB was significantly down-regulated in this study (Figure 6). Those results suggested that the suppression of NF-κB and PI3K/AKT/mTOR pathways might contribute to alisol A-induced autophagy in MDA-MB-231 cells.

Cell cycle regulating is a key method in controlling tumor propagation [44]. Recent studies suggest that, in autophagy-mediated cell death, the cell cycle is preferentially arrested in particular phases or phase transitions, such as G0/G1 or G2/M [20]. The cell cycle regulators, such as cyclin D1, cyclin B, and p21, are involved in the regulation of cell cycle [45,46]. Normal progression through G0/G1 phase of the mammalian cell cycle is dependent on the activities of Cdk4/cyclin D1 and Cdk2/cyclin E1 complexes that mediate G0/G1 phase progression [47]. In addition, p21, a negative regulator of cell cycle progression, can inhibit the activity of cyclin/Cdk2 complexes, which leads to cell cycle arrest at the G0/G1 transition [48]. Our study indicated that the expression of cyclin D1 was significantly down-regulated, while p21 was up-regulated in MDA-MB-231 cells treated with alisol A (Figure 5), suggesting the molecular mechanism through which alisol A induced G0/G1 arrest.

Furthermore, the anti-metastatic effects of alisol A was also evaluated in the present study (Figure 7A,B). Metastasis is a multi-step process that promotes cancer cell migration to distant organ sites, and many signaling pathways are involved in this process. Matrix metalloproteinases (MMPs), a family of structurally and functionally related zinc-dependent enzymes, play an important role in promoting metastasis and tumor growth. Among the MMPs, MMP-2 and MMP-9 have been involved extensively in facilitating cancer metastasis [49,50]. Our results showed that, with the treatment of alisol A, the expression of MMP-2/-9 was significantly down-regulated (Figure 7C,D). These results indicated that alisol A suppressed cell metastasis by inhibiting the activities of MMPs. Moreover, it is well-known that PI3K/Akt/mTOR and NF-κB pathways have not only been implicated in carcinogenesis but also in cancer cell invasion and the metastatic process [51,52]. The suppression of PI3K/Akt/mTOR and NF-κB pathways in MDA-MB-231 cells could be a potential molecular mechanism to elucidate the anti-metastatic effects of alisol A. Finally, recent studies have demonstrated that autophagy may exert suppressive effects in cancer metastasis [53,54,55]. However, in the present study, it is still unclear whether the anti-metastatic effects of alisol A are associated with the induction of autophagy in MDA-MB-231 cells.

## 5. Conclusions

In summary, the present study has documented the anti-proliferative and anti-metastatic effects of alisol A in MDA-MB-231 cancer cells. The inhibition of cell growth in MDA-MB-231 cells was mainly via cell cycle arrest and induction of autophagy. The effects of alisol A on cell migration and invasion inhibition might be due to the suppression of MMPs in cancer cells. These results suggest that alisol A is a potential therapeutic agent for the treatment of human breast cancer in future. However, further studies are still needed to explore its exact mechanisms and assess its therapeutic efficacy.

## Abbreviations

DMSO	Dimethyl sulfoxide
MTT	3-(4,5-dimethyl-2-thiazolyl)-2,5-diphenyl-2H-tetrazolium bromide
FBS	Fetal bovine serum
DMEM	Dulbecco’s Modified Eagle’s Medium
BSA	Bovine serum albumin
PVDF	Polyvinylidene fluoride
PBS	Phosphate buffered saline
AO	Acridine orange
PI	Propidium iodide
3-MA	3-methyladenine
TBST	Tris-buffered saline-5% Tween 20
SDS	Sodium dodecyl sulfate
ECL	Enhanced chemiluminescence
TNBC	Triple-negative breast cancer
ER	Estrogen receptor
PR	Progesterone receptor
HER2	Human epidermal growth factor receptor 2
STAT3	Signal transducer and activator of transcription 3
NF-κB	Nuclear factor-κB
